# Circulating tumor DNA in management of primary liver malignancy: A review of the literature and future directions

**DOI:** 10.1002/jso.27825

**Published:** 2024-08-19

**Authors:** Noah X. Tocci, Chase J. Wehrle, Keyue Sun, Chunbao Jiao, Hanna Hong, Abby Gross, Erlind Allkushi, Melis Uysal, Maureen Whitsett Linganna, Katheryn Stackhouse, Koji Hashimoto, Andrea Schlegel, R. Matthew Walsh, Charles Miller, David C. H. Kwon, Federico Aucejo

**Affiliations:** ^1^ Department of Hepato‐pancreato‐biliary & Liver Transplant Surgery Digestive Diseases and Surgery Institute, Cleveland Clinic Foundation Cleveland Ohio USA; ^2^ Lerner Research Institute, Inflammation & Immunity, Cleveland Clinic Foundation Cleveland Ohio USA; ^3^ Department of Gastroenterology, Hepatology, and Nutrition Digestive Diseases and Surgery Institute, Cleveland Clinic Foundation Cleveland Ohio USA

**Keywords:** cholangiocarcinoma, ctDNA, hepatocellular carcinoma, liquid biopsy, liver malignancy

## Abstract

Primary liver malignancies are a serious and challenging global health concern. The most common primary tumors are hepatocellular carcinoma and cholangiocarcinoma. These diseases portend poor prognosis when presenting with progressive, extensive disease. There is a critical need for improved diagnosis, therapeutic intervention, and monitoring surveillance in liver‐related malignancies. Liquid biopsy using ctDNA provides an opportunity for growth within these domains for liver‐related malignancy. However, ctDNA is relatively understudied in this field compared with other solid tumor types, possibly due to the complex nature of the pathology. In this review, we aim to discuss ctDNA, the current literature, and future directions of this technology within primary liver malignancies.

## 1 INTRODUCTION

Liver‐related malignancies pose a large threat to patients internationally and are a challenging global health concern. Of all cancers, liver‐related malignancies are the sixth‐most common and have a rising incidence and prevalence worldwide.^1^ Hepatocellular carcinoma (HCC) and cholangiocarcinoma (CCA) are the most common primary liver tumors. HCC accounts for the third highest rate of cancer‐related death worldwide and has a 5‐year survival that is estimated at just 18%.^1^ CCA, while more rare, also poses a high risk for mortality, with both the intrahepatic (iCCA) and extrahepatic (pCCA) forms cited to have a 5‐year survival of 10%–20%.^2^, ^3^ These malignancies often present at advanced stages, limiting treatment options, and contributing to high mortality rates.

When the disease has been detected on imaging, obtaining tissue sampling is often limited by anatomic location and complications associated with liver biopsies. Biopsies also demonstrate frequently insufficient sampling and false negative rates up to 30% and have inherent limitations in small samples capturing tumor heterogeneity and molecular evolution over time.^4^, ^5^, ^6^ Additionally, biopsy of iCCA especially raises concern for pCCA tumor seeding, thus is not preferred in most cases. Therefore, diagnosis and disease assessment primarily rely on noninvasive cross‐sectional imaging and biomarkers such as alpha‐fetoprotein (AFP), carcinoembryonic antigen (CEA), and carbohydrate antigen 19‐9 (CA 19‐9).^7^ Unfortunately, current imaging is limited in detection of disease <1 cm, and traditional biomarkers can be influenced by comorbid conditions with suboptimal sensitivity and specificity.^5^, ^8^, ^9^, ^10^ The lack of reliable biomarkers and infrequent presence of tissue samples have limited the development of precision therapeutics in both the neoadjuvant and adjuvant setting, further exacerbating the poor prognosis of these conditions.^9^, ^11^

Circulating tumor DNA (ctDNA) has become an increasingly relied upon modality for the noninvasive assessment, treatment, and monitoring of solid tumors.^11^, ^12^, ^13^, ^14^, ^15^ It is also being reported more frequently as a tool in treating liver malignancies.^7^ ctDNA are fragments of tumor DNA which freely circulate in a patient's systemic circulation, saliva and urine, allowing detection via peripheral sampling. This biomarker can provide insight into tumor biology, which can assist in diagnosis, assessment of treatment response, and screen for recurrence.^12^, ^16^, ^17^, ^18^, ^19^, ^20^, ^21^ In this narrative review, we aim to summarize the existing literature on applications of liquid biopsy in liver malignancies and discuss future directions of the field, aiming to aid both clinicians and researchers in clinical decision‐making and designing clinical studies for patients with this malignancy.^22^

## 2 CTDNA

ctDNA is composed of short segments of tumor‐derived nuclear and mitochondrial DNA freely circulating in serum, urine, saliva, bile, or feces.^12^, ^23^ The predominant theory on the mechanism for ctDNA release into patient's systemic circulation is secondary to secretion during cellular metabolism, necrosis, and apoptosis of solid tumor or circulating tumor cells.^12^, ^17^, ^23^, ^24^, ^25^, ^26^, ^27^, ^28^ ctDNA was first described in 1948, yet it was only in the 1990s that the connection was established between free‐floating tumor DNA and specific tumor biology in patients with cancer.^12^, ^17^, ^24^ Since this time, technological advancement in PCR and next‐generation sequencing has increased the feasibility of ctDNA detection.^12^, ^17^, ^29^ ctDNA has a half‐life of between minutes to a few hours, thus it serves as a near real‐time window into the cancer biology of a patient.^17^, ^30^, ^31^, ^32^, ^33^

The leading methods to isolate ctDNA is through centrifugal column and magnetic bead approaches.^22^ ctDNA can then be analyzed in either a tumor‐informed or tumor‐agnostic fashion.^14^, ^30^, ^34^ A tumor‐informed approach utilizes sequences from a patients' own tumor tissue to create PCR probes. The resulting assay is used to detect ctDNA from the patient's systemic sample. Generally speaking, tumor‐informed analysis provides a highly sensitive method to detect ctDNA while mitigating the risk of false positives.^14^ This method is thus most valuable in assessing for disease recurrence, response to treatment, and evaluation of minimal residual disease.^14^, ^35^, ^36^ Conversely, tumor‐agnostic analysis does not require tissue sampling of the tumor and is most useful for comprehensive genomic profiling. There is also an additional critical benefit that tumor agnostic sequencing does not require tissue sampling. As mentioned previously, it is quite challenging to obtain such samples in many liver patients without major surgery, which limits tumor‐informed sequencing in this population. Tumor agnostic sequencing allows for discovery of common genomic anomalies that develop through tumor evolution, which might be an indication of tumor therapeutic resistance.

While ctDNA can detect as few as single molecules of DNA in a sample as small as 100 μL, ensuring adequate sensitivity and specificity requires background signal from normal cells to be diminished while amplifying the ctDNA.^16^, ^17^ To this end, amplification and parallel sequencing techniques such as BEAMing, Droplet Digital™ PCR, TEC‐Seq, CancerSeek have been developed, yielding a much higher detection rate versus traditional PCR.^17^ Additionally, while blood samples are the most frequent source of sampling for ctDNA, other non‐blood specimens such as peritoneal fluid, bile, stool have been preliminarily studied and may contain even higher levels of ctDNA.^14^, ^16^, ^37^, ^38^, ^39^ Overall, the improving technology for ctDNA has allowed it to gain clinical use in various malignancies such as breast, non‐small cell lung cancer, pancreatic, gastrointestinal stromal tumors, HCC, CCA, and colorectal cancers.^17^ Of note, the National Comprehensive Cancer Network has even instituted ctDNA sampling into practice guidelines for non‐small cell lung cancer^40^ and numerous healthcare policies are covering the use of ctDNA testing.^41^

## 3 HEPATOCELLULAR CARCINOMA (HCC)

HCC is the third leading cause of cancer‐related deaths in the world and is responsible for 90% of all primary liver malignancies.^1^, ^3^ AFP is currently the only biomarker that has been studied and approved for screening and diagnosis of HCC.^1^, ^42^, ^43^ Despite the utilization of AFP for detection and screening for HCC, it is a very imperfect biomarker with a sensitivity for early HCC of 39%–64%, specificity of 76%–97%, and positive predictive values of 25% at 5% disease prevalence.^1^, ^9^, ^43^, ^44^ AFP levels are heavily influenced by various comorbid states, and 40%–50% of HCC do not present with elevated AFP levels.^13^

With current standard biomarker evaluations of HCC proving to be inadequate, a large interest has grown to find a more suitable window into a patients' current tumorigenic state. The first relevant utility of ctDNA is the potential for early detection of disease. One study sought to compare AFP with ctDNA in HCC patients and found that liquid biopsy identified 70% of patients with HCC, whereas AFP only positively identified 56.8% of HCC.^45^ Further supporting this utility, Su et al. found that when testing samples of AFP‐negative patients, ctDNA was positive in 92% of these samples.^13^ While ctDNA has been linked to tumor size, angiogenesis of the tumor, and ctDNA shedding levels, there has been some discrepancy as to what minimum tumor size is required for ctDNA detection. Early reports show that ctDNA can be detected 4 months before detection of disease recurrence on traditional imaging, and others note that ctDNA becomes detectable at >1 cm which would be picked up by standard imaging.^4^, ^46^

Liquid biopsy has detected genetic alterations specific to HCC in the Wnt signaling (incidence 13%–15% –80%–90%), cell cycle and apoptosis (20%–50% – 55%–85%), telomere maintenance (29%–59%), mTOR signaling (16%–43%), chromatin remodeling (5%–18%), JAK/STAT (50%–93%), detoxification (38%–80%), and protease inhibitor (47%) pathways (Table 1).^13^ Given its ability to identify these targetable pathways, liquid biopsy can provide an avenue to tailor therapeutic interventions to the particular biology of the patient and combat evolving tumor drug resistance.^78^ The DYNAMIC trial, which looked at ctDNA‐guided adjuvant therapy for early‐stage colorectal patients, showed that it is feasible to de‐escalate adjuvant chemotherapy without increasing the risk of recurrence‐free survival.^79^ This work may provide a foundation for future ctDNA‐guided treatments for HCC patients.

**Table 1:** Summary of genetic pathways in HCC and the corresponding clinical relevance.

Gene/genetic pathway	Study	ctDNA positivity	Relevance
Telomere maintenance	[1, 44, 47, 48]	Yes	‐ Telomere reverse transcriptase (TERT) is a mutation that affects telomere maintenance. ‐ One of the most frequent mutations in HCC with 60% of HCC containing this mutation. ‐ ctDNA has 87% sensitivity for detection of HCC in chronic Hepatitis C patients through TERT promoter detection.
Cell cycle/apoptosis	[4, 49, 50, 51, 52, 53, 54, 55, 56, 57, 58]	Yes	‐ RASSF1A—regulates cell cycle and triggers apoptosis in addition to microtubule stability, seen in 70% of all malignancies. ctDNA positive detection in 50‐90%. ‐ CDKN2A (pl6) mutation occurs 50%–70% of HCC; associated with poorer overall survival and disease‐free survival. It serves as a prognostic biomarker correlating with immune infiltrates in HCC. ctDNA detection with a sensitivity of 44‐92% in serum and a specificity of 68%–96%. ‐ p53‐ associated with chromosomal instability, and mutations occur in 20%–50% of HCC. ctDNA detection rate ~50%– 90%.
Wnt signaling	[13, 50, 59, 60, 61, 62, 63, 64, 65]	Some mutations	‐ Alterations in Wnt pathway can initiate cell proliferation through modulation of beta‐catenin. ‐ Genes that are commonly altered in this pathway in HCC are APC, CTNNB1, AXIN1 and SFRP1 genes. 75% of HCC contains mutations in this pathway. ‐ APC mutations or mutations to the promoter region are seen in ~50%–80% of HCC. ‐ SFRP1 forms an inhibitory complex which inhibits Wnt pathway and is a negative regulator of cell invasion which could possibly predict the metastatic potential.
mTOR signaling	[66, 67, 68, 69]	Some mutations	‐ Play a regulatory role in proliferation, migration and angiogenesis. ‐ PTEN‐ not detected by ctDNA liquid biopsy. ‐ mRASSF1A serum ctDNA detection with sensitivity 70%–100% and specificity 52%–100%.
Chromatin remodeling	[68, 70, 71, 72]	No	‐ 50% of HCC cases have mutations (ARID1A, ARID1B and ARID2). ‐ These mutations are lead to increased cell proliferation.
JAK/STAT	[50, 63, 73]	No	‐ Pathway responsible for cell proliferation, migration, and angiogenesis ‐ Highly prevalent (45%) in HBV and HCV‐associated HCC.
Detoxification	[50, 74, 75, 76, 77]	Yes	‐ mGSTP1 with ctDNA serum detection sensitivity at 50% and specificity of 80%.
Protease inhibitor	[76, 77]	Yes	‐ mTRP12 with a ctDNA serum detection sensitivity of 47% and specificity of 80%.
VEGF‐A gene amplifications	[1]	Yes	‐ Improved therapeutic response with VEGF‐A gene amplification present to sorafenib therapy.

Abbreviation: HBV, hepatitis B virus; HCC, hepatocellular carcinoma; HCV, hepatitis C virus; VEGF, vascular endothelial growth factor.

In this vein, recent work by Wehrle et al demonstrated that tumor mutational burden (TMB) from ctDNA can predict recurrence after curative‐intent resection with a negative predictive value (NPV) = 90%.^80^ The authors theorize this is due to the ability of ctDNA to predict recurrence rather than identify it. This work provides initial promising evidence supporting the use of TMB/ctDNA for surveillance, however, it was a small sample size (*n* = 47), and this work needs validation before widespread clinical use. There has been significant work showing that TMB detected in tissue samples can predict response to immunotherapy.^49^, ^81^, ^82^ This knowledge coupled with the impressive NPV of TMB in the Wehrle et al study indicates it is reasonable to consider how liquid biopsy‐based TMB might be able to guide specifically the de‐escalation of immunotherapy, which has clearly emerged as the preferred systemic treatment for HCC in the adjuvant setting.^83^, ^84^, ^85^ This is the same approach used in the DYNAMIC trial for CRC, though this would of course require prospective and randomized data before clinical implementation.

In addition to guiding systemic therapy, ctDNA has been shown to be a useful prognostic tool in HCC. Wang et al. found that in patients undergoing resection of HCC, ctDNA status was only second to the Barcelona Clinic Liver Cancer (BCLC) stage in predicting postoperative recurrence risk.^45^ They also found that positive preoperative ctDNA status was associated with larger tumor sizes, multiple lesions, micro‐vascular invasion, shorter disease‐free interval, and lower overall survival of patients.^45^ Raj et al. utilized ctDNA to assess patient response to immunotherapy plus local regional therapy with subsequent hepatectomy for curative intent with three of the four patients notably clearing ctDNA status curative intent surgery with extended follow up >40 months having no notable evidence of disease.^86^ This team also has established institutional protocol for ctDNA‐based surveillance, providing guidance to other programs looking to integrate such technology in complex multidisciplinary surgical practice. Finally, the same group (reported by Hong et al.) reported an experience with ctDNA before and after liver transplantation for HCC, CCA, and CRLM, noting clearance of ctDNA after transplantation in many patients and noting higher recurrence rates in those with postoperative ctDNA positivity.^87^

This experience does also report the first insights into the potential for donor‐derived DNA, where tissue‐agnostic sequencing may identify mutations from the donor of the solid organ transplant. This could represent a limitation of tissue‐agnostic sequencing in the postoperative setting. However, tissue‐informed sequencing is technically unfeasible before transplant, and this same study noted a preliminary correlation between preoperative ctDNA positivity and postoperative disease recurrence, highlighting the utility of tissue agnostic sequencing before transplantation.^87^

With growing interest for early detection, personalized therapy, recurrence surveillance, and prognostic data, liquid biopsy through detection of ctDNA for HCC has increasing interest and utilization in the clinical space. Future directions should aim to increase utilization of this tool in a systematic approach in the clinical setting.

## 4 CHOLANGIOCARCINOMA

CCA comprises 3% of all gastrointestinal tumors and is the second‐most common cancer of the liver.^88^ CCA has traditionally been classified by tumor site of origin within the biliary ducts, delineated by iCCA, perihilar (pCC), and eCC.^88^, ^89^ Sufficient tissue is generally preferred to assess gene alterations before selecting therapeutic intervention.^90^ This is, however, challenging, as both iCCA and pCCA are often prohibitively located for obtaining an adequate tissue sample. As such, biomarkers have been utilized for assistance in detection and tracking of treatment response to CCA but are very limited in this context. CA 19‐9 is the only recommended biomarker for clinical use according to ESMO guidelines.^91^ Despite this recommendation, the clinical meaning of Ca19‐9 levels is controversial given its lack of overall specificity due to overlap with other malignancies, as well as benign cholestatic and hepatic injuries.^14^, ^88^, ^90^, ^92^ Rizzo et al. reviewed the current literature of CA19‐9 and CEA levels in the diagnosis CCA and found that the sensitivity of these biomarkers were 77.14% and 68.57% respectively, with CA19‐9 having a false positive rate of 15.22% and CEA false positive rate of 18.48%.^92^ In a review published by Mody and Cleary, they cite that only 60%–65% of CCA are associated with elevated CA 19‐9.^88^ Therefore, current biomarkers for CCA are frequently not useful for disease monitoring, and further their assessment of tumor biology is helpful in some cases, but frequently quite limited.

Given these limitations, liquid biopsy has also gained relevance in this condition. For example, the presence ctDNA levels above 0.2175 and 0.3388 ng/µL yielded a sensitivity for CCA of 88.7 versus 82.3% for healthy controls, and also identified patients with benign disease, with a specificity of 96.7% and 57.6%, respectively.^93^ When comparing a heterogenous group of patients with a mix of iCCA and eCCA, ctDNA had an 89% sensitivity and 97% specificity in diagnosing disease states, which outperformed both CEA and CA19‐9.^90^ Additionally, the accuracy of liquid biopsy unsurprisingly increases with bulk of disease and metastatic disease. ctDNA has been detected in 90% of metastatic CCA disease, and detected clonal evolution at disease progression, thus allowing it to be a useful tool for assessing resistant disease.^14^ Wintachai et al. noted that levels of ctDNA corresponded with tumor size, nodal metastasis, and tumor staging, thus supporting the notion that ctDNA correlates with disease severity and progression and therefore may offer prognosticating value.^93^

The anatomic sites of CCA have variable tumor genetics, and this has implications for therapeutic targeting. ctDNA has been able to detect somatic mutation in genes of iARID1A, IDH1, KRAS, PBRM1, MTOR, FGFR, TP53, PTEN, NCOR1, EPHA2, PIK3CA, TERT, RASA1, EZH2, and BAP1.^93^ Mutations such as IDH1 (15%–20% of cases) and FGFR2 fusions (10%–20%) are the most prevalent alterations in CCA with Food and Drug Administration (FDA) approved targeted therapies (Table 2).^90^ This is particularly relevant in iCCA disease since these alterations of IDH1 and FGFR2 fusions are almost exclusively seen in iCCAs, and ctDNA levels have been used to both monitor treatment response, as well as detect tumor resistance mechanisms.^20^, ^92^, ^93^, ^94^ Okamura et al. sought to investigate the efficacy of targeted therapeutic approaches using ctDNA for biliary tract cancer patients and found that 70% of alterations were targetable by FDA‐approved agents, and with those patients who received molecularly matched therapeutic regimens based off the ctDNA profile, had an improved progression‐free survival and higher disease rate control compared to unmatched regimens.^94^

**Table 2:** Summary of genetic pathways in CCA and the corresponding clinical relevance.

Gene/genetic pathway	Study	ctDNA positivity	Relevance
IDH1	[94, 95, 96]	Yes	‐ Mutation in this gene affects cell cycle regulation. ‐ 10%–15% of patients with CCA found to have this mutation. ‐ This is almost exclusively seen in iCCA. ‐ FDA‐approved targeted therapy for this mutation, ivosidenib. ‐ Mutations to this gene can be used to monitor for tumor resistance.
FGFR2	[94, 95, 96, 97, 98]	Yes	‐ Mutations in this gene affect cell growth and regulation. ‐ Seen in 14% of CCA cases. ‐ FDA‐approved targeted therapies include pemigatinib, infigratinib, and futibatinib for FGFR2
TP53	[93, 94, 95, 96]	Yes	‐ Mutations in this gene affect tumor suppression and apoptotic pathways. ‐ 38%–44% of patients with CCA found to have this mutation. ‐ ctDNA was detected in 68% of patients with this mutation.
KRAS	[92, 94, 95, 96]	Yes	‐ Mutations in this gene affect cell signaling pathways which affect cell growth, maturation, and apoptosis ‐ 28%–40% of patients with CCA found to have this mutation with ctDNA detecting this mutation in 80% of patients.
CDKN2A/B	[94, 95, 96]	Yes	‐ Mutations in this gene affect tumor suppression ‐ 33% of patients with CCA found to have this mutation ‐ This mutation has poor ctDNA detection of only 3%
PIK3CA	[93, 94, 95, 96]	Yes	‐ Mutations in this gene affect cell proliferation ‐ 14% of patients with CCA found to have this mutation with ctDNA detecting 90% of these mutations
BRCA2	[10, 94, 95, 96]	Yes	‐ Mutations in this gene affect DNA repair. ‐ Pathogenic mutation in BRCA2 occurs in 4.3% of CCA patients ‐ This population has seen response to PARP inhibitors

Abbreviation: CCA, cholangiocarcinoma

## 5 CONCLUSION

**Figure 1:**
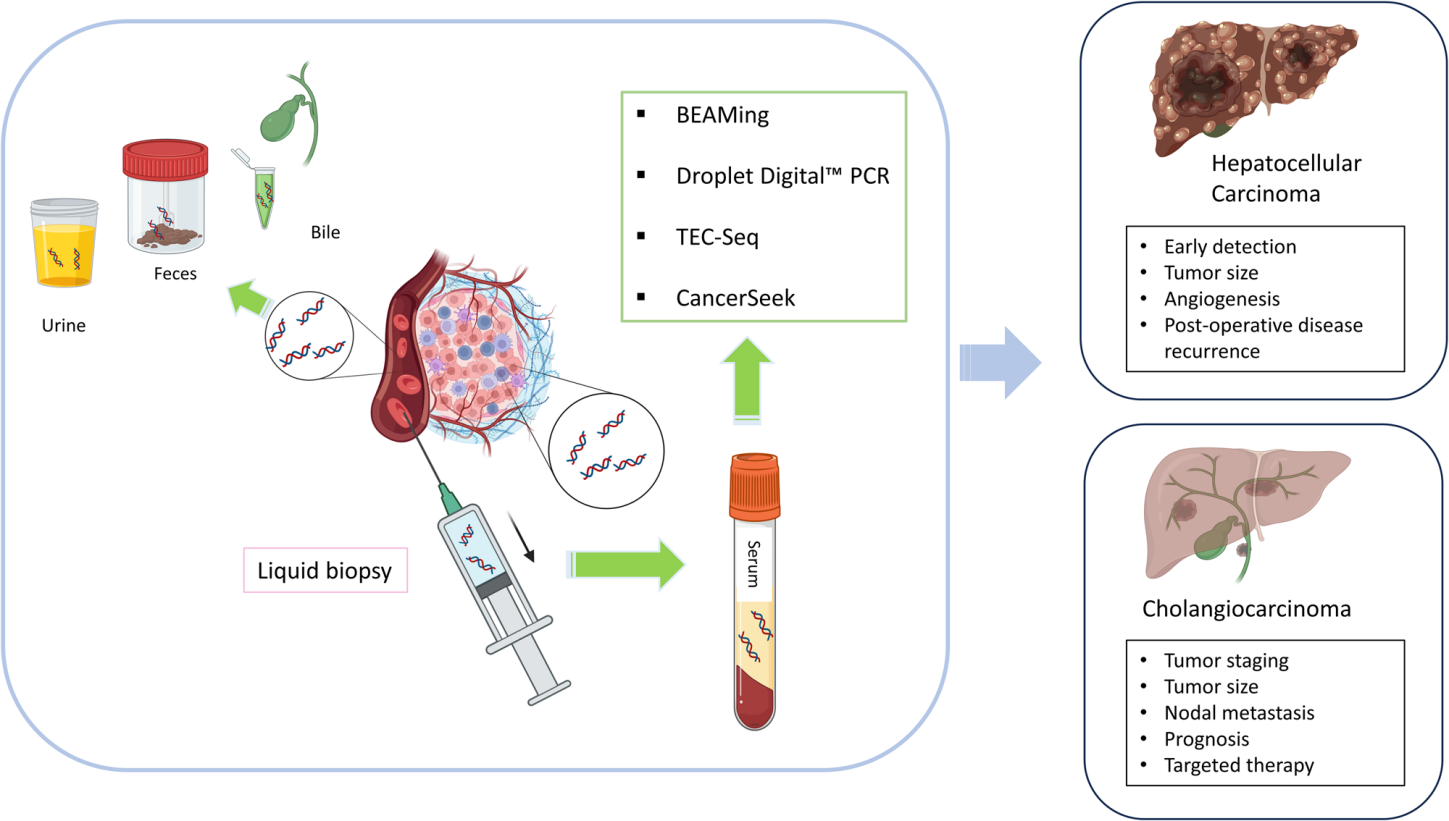
ctDNA‐based Liquid Biopsy in Primary Liver Cancers.

The evolving landscape of liquid biopsy of ctDNA has shown great promise and still has ample opportunity for growth in the clinical space. ctDNA has exhibited value as an adjunct to current biomarkers for screening and surveillance, and with regard to therapeutic selection, minimal residual disease recurrence, and longitudinal monitoring. Future work is necessary on feasibility of implementation of ctDNA into clinical practice. Perhaps most critically, randomized and prospective trials are needed in the clinical management of primary liver and other gastrointestinal cancers using ctDNA (Figure 1).

## AUTHOR CONTRIBUTIONS

The study was conceptualized and conducted under the direction of Dr. Federico Aucejo and Dr. David Kwon. Literature review and analysis was performed by Noah X. Tocci, Chase J. Wehrle, and Hanna Hong. Manuscript drafting was performed by Noah X. Tocci, Chase J. Wehrle and Abby Gross. Critical manuscript review was performed by all authors.

## CONFLICT OF INTEREST STATEMENT

The authors of this article have no conflict of interest or financial disclosures to report.

## SYNOPSIS

Circulating tumor DNA has become a critical component of the management of many solid‐organ malignancies. However, it remains relatively under described in primary liver cancers. We describe the utility of ctDNA in the pre‐ and postoperative management of primary liver cancer, focusing on hepatocellular and cholangiocarcinoma, with an additional focus on future perspectives.

## Data Availability

All data is publicly available (this is a review of the literature).
